# Serum Anti-PDLIM1 Autoantibody as Diagnostic Marker in Ovarian Cancer

**DOI:** 10.3389/fimmu.2021.698312

**Published:** 2021-08-19

**Authors:** Cuipeng Qiu, Yaru Duan, Bofei Wang, Jianxiang Shi, Peng Wang, Hua Ye, Liping Dai, Jianying Zhang, Xiao Wang

**Affiliations:** ^1^Henan Institute of Medical and Pharmaceutical Sciences, Zhengzhou University, Zhengzhou, China; ^2^State Key Laboratory of Esophageal Cancer Prevention and Treatment & Henan Key Laboratory of Tumor Epidemiology, Zhengzhou, China; ^3^School of Basic Medical Sciences, Academy of Medical Sciences, Zhengzhou University, Zhengzhou, China; ^4^Department of Epidemiology and Health Statistics, College of Public Health, Zhengzhou University, Zhengzhou, China

**Keywords:** autoantibody, tumor-associated antigens, ovarian cancer, PDLIM1, diagnostic marker

## Abstract

**Background:**

Serum autoantibodies (AAbs) against tumor-associated antigens (TAAs) could be useful biomarkers for cancer detection. This study aims to evaluate the diagnostic value of autoantibody against PDLIM1 for improving the detection of ovarian cancer (OC).

**Methods:**

Immunohistochemistry (IHC) test in tissue array containing 280 OC tissues, 20 adjacent tissues, and 8 normal ovarian tissues was performed to analyze the expression of PDLIM1 in tissues. Enzyme-linked immunosorbent assay (ELISA) was employed to measure the autoantibody to PDLIM1 in 545 sera samples from 182 patients with OC, 181 patients with ovarian benign diseases, and 182 healthy controls.

**Results:**

The results of IHC indicated that 84.3% (236/280) OC tissues were positively stained with PDLIM1, while no positive staining was found in adjacent or normal ovarian tissues. The frequency of anti-PDLIM1 autoantibody was significantly higher in OC patients than that in healthy and ovarian benign controls in both training (n=122) and validation (n=423) sets. The area under the curves (AUCs) of anti-PDLIM1 autoantibody for discriminating OC from healthy controls were 0.765 in training set and 0.740 in validation set, and the AUC of anti-PDLIM1 autoantibody for discriminating OC from ovarian benign controls was 0.757 in validation set. Overall, it was able to distinguish 35.7% of OC, 40.6% of patients with early-stage, and 39.5% of patients with late-stage. When combined with CA125, the AUC increased to 0.846, and 79.2% of OC were detected, which is statistically higher than CA125 (61.7%) or anti-PDLIM1(35.7%) alone (*p*<0.001). Also, anti-PDLIM1 autoantibody could identify 15% (18/120) of patients that were negative with CA125 (CA125 <35 U/ml).

**Conclusions:**

The anti-PDLIM1 autoantibody response in OC patients was positively correlated with PDLIM1 high expression in OC tissues, suggesting that the autoantibody against PDLIM1 might have the potential to be a novel serological biomarker of OC, serving as a complementary measure of CA125, which could improve the power of OC detection.

## Introduction

Ovarian cancer (OC) is one of the most common cancer among women, with approximately 313,959 new diagnoses and 207,252 deaths worldwide in 2020 (1). Due to the typically asymptomatic early-stage tumors and lack of effective diagnostic methods, most OC patients continue to be diagnosed at advanced stage with high fatality rates and relatively poor long-term survival (2, 3). Although some improvements in diagnosis and treatment have been made, the overall 5-year survival rate still remains as low as 40% (4). The measurement of CA125 level has been embraced by primary care for ovarian cancer, but the measurements with better sensitivity and specificity are especially in need for early disease (5). To address this issue, substantial work needs to be put into the exploration of novel biomarkers that could improve early diagnosis and treatment effect.

The detection of autoantibodies (AAbs) triggered by tumor-associated antigens (TAAs) is showing great potential for the development of blood-based biomarkers (6, 7). Most of TAAs are secreted or shed into the blood from tumor cells, then captured by the immune system and elicit an immune response (8). However, due to the labile features and low titers, TAAs seem not as stable as AAbs. AAbs not only have an immunological amplification effect but also can exist in the peripheral blood for a longer period of time, making them more ideal biomarkers for the detection of cancers than their corresponding TAAs (9). Moreover, AAbs have been identified as the reporters of incipient carcinogenesis, which could occur before any clinical symptoms (10). Therefore, identifying AAbs with good performance will hold favorable clinical applications, especially for the early detection of cancer with a non-invasive method.

The cytoskeleton is a kind of protein fiber network structure and widely exists in eukaryotic cells, which maintains cell morphology and participates in cell movement, cell polarity, cell division, and signal transduction (11). PDLIM1 is a cytoskeletal protein, also known as CLP36, belonging to the PDZ and LIM protein family. Mediated *via* the PDZ domain, PDLIM1 binds to alpha-actinin and is localized to actin stress fibers in the cytoplasm (12). Many studies demonstrated that PDLIM1 plays a role in the regulation of actin cytoskeleton organization in non-muscle tissues (12–14). Studies indicated that the abnormal expression of PDLIM1 is associated with hepatocellular carcinoma, breast cancer, colorectal cancer, and pancreatic cancer (15–18). In Liu’s study, PDLIM1 was reported to promote breast cancer cell migration and invasion *in vitro* and metastasis *in vivo* through interaction with α-actinin (17). During metastasis of hepatocellular carcinoma (HCC), PDLIM1 was demonstrated to play an important inhibitory role through activating the Hippo signaling pathway (16). It seems that PDLIM1 plays different regulatory roles in different kinds of cancer cells (16, 17).

In a few studies on autoantibody against PDLIM1, PDLIM1 was identified as a tumor-associated antigen (TAA) due to inducement of autoantibody response in patients with breast cancer and pancreatic cancer (9, 18). No reports have been found about the expression of PDLIM1 in ovarian cancer tissues and whether there is an autoantibody response to PDLIM1 in patients with ovarian cancer. In the current study, we aim to explore the occurrence and presentation level of anti-PDLIM1 autoantibodies in the sera of patients with ovarian cancer and further to evaluate its potential as a biomarker for the detection of OC.

## Materials and Methods

### Immunohistochemistry

OC tissue microarray consisting of tissues from 294 OC patients (14 of 294 were invalid), 20 adjacent tissues, and 8 normal ovarian tissues was obtained from Shanxi Avila Biotechnology Ltd., Co. (Xian, China), and duplicate cores per case of cancer to make sure of a solid result. Immunohistochemistry (IHC) test was performed by following the standard protocols. Anti-PDLIM1 antibody (Santa Cruz Biotechnology, sc-374077, 1:20 dilution) and secondary antibody (MXB Biotechnologies, KIT-9720) were used for IHC testing. Briefly, paraffin-embedded tissue slides were deparaffinized and rehydrated with xylene and graded alcohols. Slides were washed with phosphate-buffered saline (PBS) and subjected to antigen microwave retrieval at 100°C for 15 min and cooled at room temperature for 40 min. Endogenous peroxidase activity was blocked by incubating slides with 3% H_2_O_2_ for 37°C/30 min. Slides were blocked by 10% blocking reagent (goat serum) for 60 min. After washing with PBS, slides were incubated with PDLIM1 antibody at 4°C overnight. Slides were then incubated with secondary antibody for 60 min at room temperature. To visualize the reaction, slides were incubated with DAB for 2–5 min at room temperature and followed by counterstaining with Gill hematoxylin solution for 1 min and washed for 10 min with running water. Finally, the slides were dehydrated and mounted and were then observed under a microscope (Olympus). The results were read by two independent pathologists. Stain intensity and the percentage of positive cells were scored as follows: (1) for stain intensity, negative, score 0; light brown, score 1; brown, score 2; deep brown, score 3; (2) for percentage of positive cells, scored each criterion on a scale of 0 to 3, ≤5% scored 0; 6–25% scored 1, 26–50% scored 2, and >50% scored 3. For two cores per case of cancer, the result was calculated by the mean value. Final results (defined as IHC-score) were calculated by multiplying the scores of the percentage of positive cells by the stained intensity. The range of IHC-score is 0–9, and if it was greater than 2, the sample was considered as positive result (19). The expression of PDLIM1 in OC and normal ovarian tissues was also explored at the Human Protein Atlas (HPA) (20).

### Sera From Patients and Controls

The case-control study including 545 subjects were divided into two cohorts. All female OC cases were age-matched with corresponding healthy controls. Healthy controls had no history of cancer, autoimmune diseases, and ovarian benign diseases. The 182 OC sera and 181 benign controls sera were obtained from two affiliated hospitals of Zhengzhou University in Henan Province, China, and serum collection time spanned July 2017 to December 2018. All healthy control sera (N=182) were from the Biological Specimen Bank of Henan Key Laboratory of Tumor Epidemiology. The detailed information of the study populations is shown in **Table 1**. All participants in the study have signed the informed consent form. The study was approved by the Ethics Committee of Zhengzhou University.

**Table 1:** Characteristics of study subjects.

Variables	Training set		Validation set		
	OC (%)	Healthy (%)	OC (%)	Benign (%)	Healthy (%)
Number	61	61	121	181	121
Female	61 (100)	61 (100)	121 (100)	181 (100)	121 (100)
Age, years					
Mean ± SD	54 ± 10	51 ± 12	51 ± 12	40 ± 11	52 ± 11
Range	23–74	23–81	20–81	22–66	20–83
Family tumor history				NA	NA
Yes	20 (32.8)		24 (19.8)		
No	41 (67.2)		97 (80.2)		
FIGO				NA	NA
I	6 (9.8)		15 (12.4)		
II	2 (3.2)		9 (7.4)		
III	19 (31.1)		32 (26.4)		
IV	10 (16.4)		20 (16.5)		
Missing	24 (39.3)		45 (37.2)		
Lymph node metastasis				NA	NA
Positive	26 (42.6)		38 (31.4)		
Negative	35 (57.4)		83 (68.6)		
Distant metastasis				NA	NA
Positive	25 (41.0)		34 (28.1)		
Negative	36 (59.0)		87 (71.9)		
Histological type				NA	NA
Serous adenocarcinoma	51 (42.1)		102 (84.3)		
Mucinous adenocarcinoma	1 (0.8)		3 (2.5)		
Clear cell carcinoma	2 (1.7)		3 (2.5)		
Endometrioid adenocarcinoma	7 (5.8)		13 (10.7)		

OC, ovarian cancer.

NA, not applicable.

### Recombinant Protein and ELISA Assay

The full-length recombinant protein of PDLIM1 was purchased from Cloud-Clone Corp. (Wuhan, China). PDLIM1 recombinant protein was diluted in carbonate buffer (pH=9.6) to an optimal concentration of 0.25 μg/ml for ELISA testing. The detailed procedure was described in our previous study (21). In brief, the diluted protein was coated onto the bottom of 96-well plates overnight at 4°C, followed by incubation using 2% bovine serum albumin (BSA) for 2 h at 37°C water baths. After washing with phosphate-buffered saline containing 0.05% Tween-20 (PBST), sera with the dilution of 1:100 or the dilution buffers without sera (blank control) were added into corresponding wells for incubation of 1 h at 37°C water baths. In this step, eight sera from four OC patients and four healthy controls were added into every plate for normalization among different plates (CV<15%). Then, plates were washed by PBST followed by incubating with HRP-conjugated goat anti-human IgG at 1:5m000 dilution for 1 h at 37°C water baths. A solution of 3,3’,5,5’-tetramethyl benzidine (TMB)-H_2_O_2_-urea was used as the detecting agent, and 2M sulfuric acid was added into each well as the stopping solution. The optical density (OD) was read at 450 and 620 nm by Multilabel Plate Reader (PerkinElmer).

### Statistical Analysis

All data were described by using Median ± IQR (Inter Quartile Range). Kruskal-Wallis H Test, Mann-Whitney U Test, Chi-square test, and Fisher’s Exact Test were performed to compare the differences of AAb levels in different groups (if there were more than two groups for comparison, the α value was adjusted by Bonferroni correction). The receiver operating characteristics (ROC) curve analysis was employed to evaluate the diagnostic value of anti-PDLIM1 AAb for OC. In addition, the Youden index (YI), sensitivity, specificity, false positive rate (FPR), false negative rate (FNR), positive predictive value (PPV), and negative predictive value (NPV) were calculated to evaluate the validity and reliability of the anti-PDLIM1 AAb as a diagnostic biomarker. The cut-off value was determined by the maximum of Youden index with specificity of 90%. Statistical analyses were performed by IBM SPSS Statistics 21.0 and GraphPad Prism 8.0. The gene expression of PDLIM1 in OC tissues was investigated in GEPIA (Gene Expression Profiling Interactive Analysis) (22).

## Results

### PDLIM1 Protein Expression in OC Tissues

**Figure 1:**
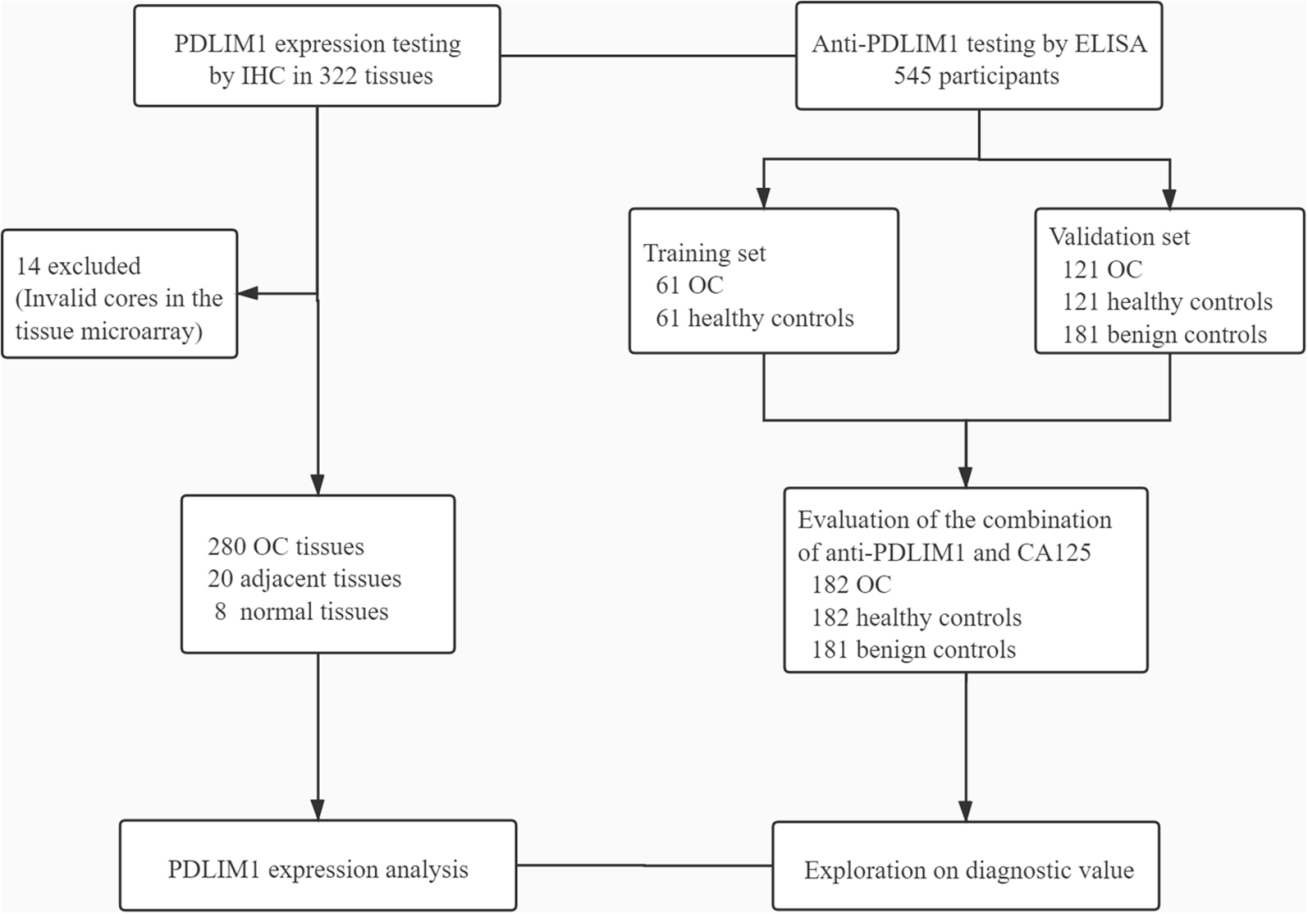
Study design. OC, ovarian cancer; ELISA, enzyme-linked immunosorbent assay; IHC, immunohistochemistry.

**Figure 2:**
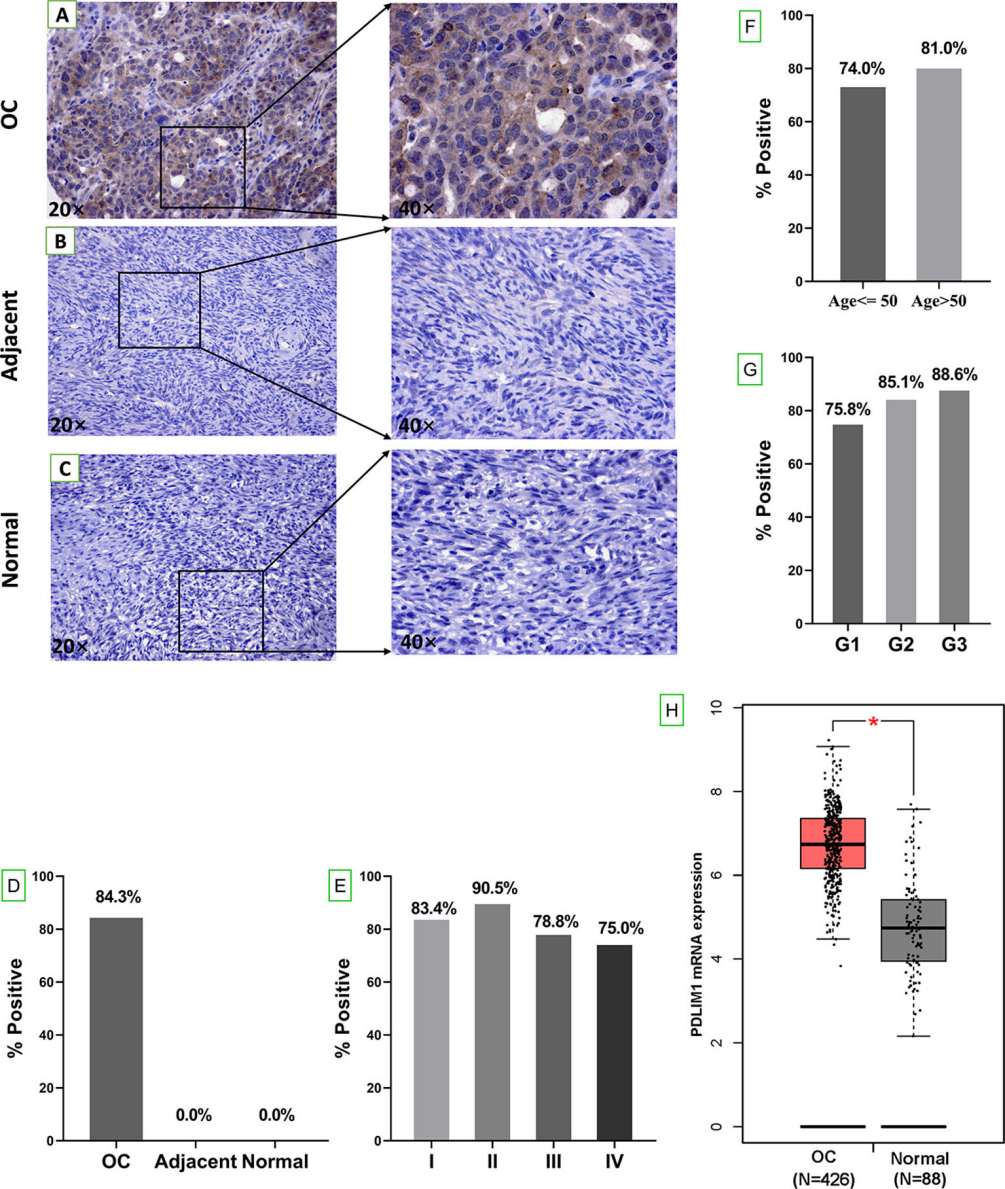
Tissue expression of PDLIM1 by IHC analysis. **(A)** Positive staining of PDLIM1 in a representative ovarian cancer tissue (obtained at 20× and 40× by microscope). **(B)** Negative staining in a representative adjacent tissue (obtained at 20× and 40× by microscope). **(C)** Negative staining in a representative normal ovarian tissue (obtained at 20× and 40× by microscope). **(D)** Positive rates of PDLIM1 in the tissues of 280 OC, 20 adjacent normal tissues, and 8 normal ovarian tissues. **(E)** Positive rates of PDLIM1 in the different stages of 280 OC. **(F)** Positive rates of PDLIM1 in different ages. **(G)** Positive rates of PDLIM1 in different pathological grades. **(H)** The mRNA expression of PDLIM1 in OC and normal tissues from GEPIA database. *p < 0.05. The cut-off value was considered as IHC-score >2.

The overall study design was shown in **Figure 1**. The expression of PDLIM1 protein was tested and analyzed in OC tissues, adjacent normal tissues, and normal ovarian tissues by IHC (**Table 2**). According to the IHC results, PDLIM1 was highly expressed in OC tissues (**Figure 2A**) with cytoplasmic staining pattern, while no cytoplasmic staining was found in both adjacent tissues and normal ovarian tissues (**Figures 2B, C****)**. There were 84.3% (236/280) of OC tissues (14 of 294 cores were invalid) that were positively stained with PDLIM1 (**Figure 2D**). Based on clinical features such as clinical stage, age, and pathological grade, OC tissues were classified into three subgroups. Across the three subgroups, there were no significant differences in frequency of positive staining (**Figures 2E** **–G**). By querying the HPA database, it was found that the expression of PDLIM1 had strong or weak staining (8 of 11) in OC tissues, while normal ovarian tissues showed negative staining in ovarian stromal cells. Moreover, we also analyzed the mRNA expression of PDLIM1 in OC and normal ovarian tissues by GEPIA; it was also highly expressed (*p*<0.05) in OC tissues with significant difference (**Figure 2H**).

**Table 2:** Results of IHC analysis.

Tissues	Stages	N	Intensity score		Positive-cell score		IHC-Score	
			Median	IQR	Median	IQR	Median	IQR
OC	I	193	2.0	1.0	3.0	1.0	6.0	6.5
	II	42	2.0	1.0	3.0	0.0	6.0	6.0
	III	33	1.0	1.0	3.0	1.0	3.0	4.0
	IV	12	2.5	1.8	3.0	1.1	7.5	6.4
	Total	280	2.0	1.0	3.0	1.0	6.0	6.4
Adjacent		20	0.0	0.0	0.0	0.0	0.0	0.0
Normal		8	0.0	0.0	0.0	0.0	0.0	0.0

OC, ovarian cancer; IQR, interquartile range; IHC, immunohistochemistry.

### Detection and Validation of Anti-PDLIM1 Autoantibody by ELISA

**Figure 3:**
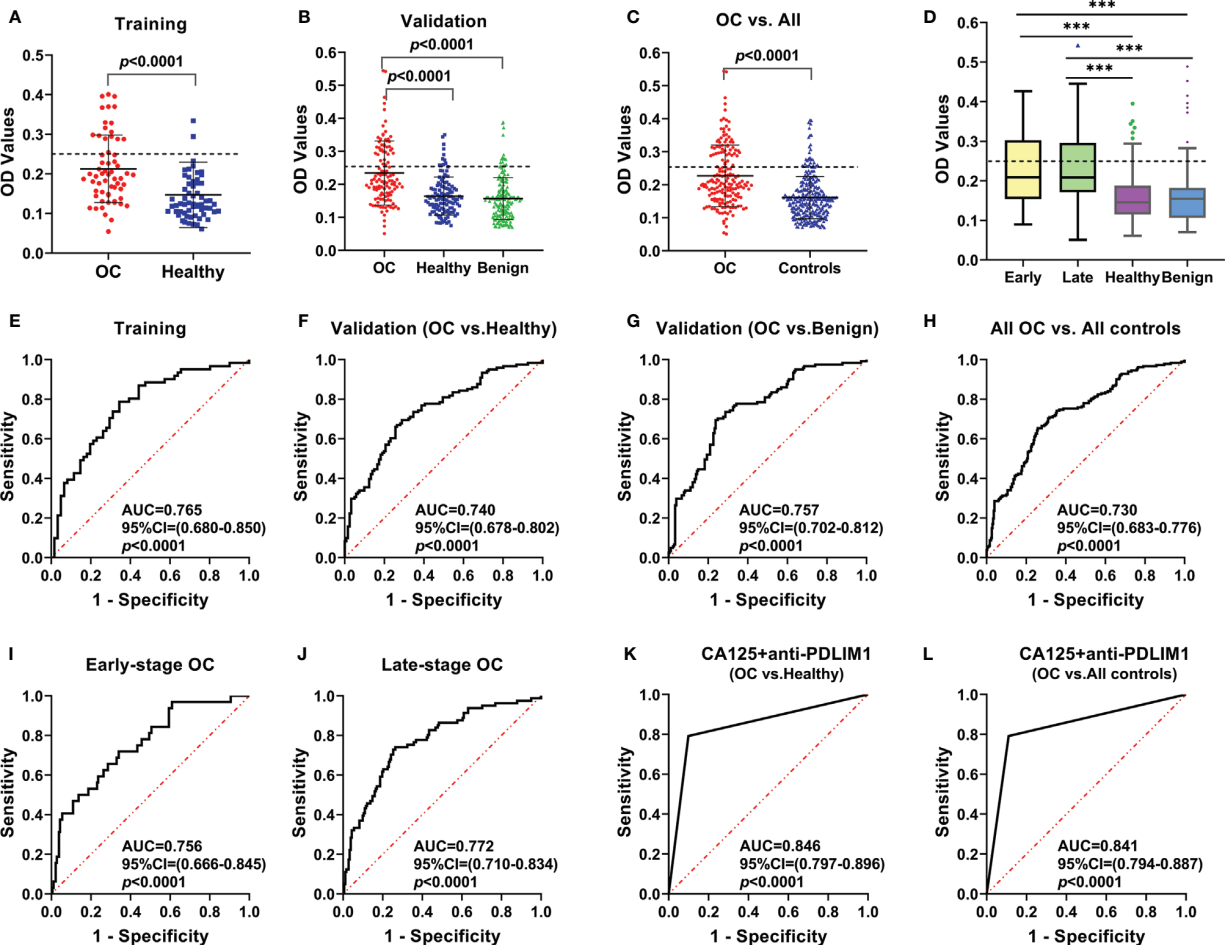
The expression and the diagnostic performance of anti-PDLIM1 AAb in OC. The expression level of anti-PDLIM1 AAb in the training set **(A)**, validation set **(B)**, all OC and all controls (healthy+benign) **(C)**, early-stage and late-stage of OC **(D)**. The cut-off value (dotted line) was determined by the maximum Youden index with the specificity of 90%. The diagnostic performance of anti-PDLIM1 AAb in the training set **(E)**, validation set **(F, G)**, all OC and all controls (healthy+benign) **(H)**, early-stage OC **(I)**, late-stage OC **(J)**. The diagnostic performance of anti-PDLIM1 AAb in combination with CA125 for identifying OC from healthy controls **(K)** or all controls (healthy+benign) **(L)**. ***p < 0.001.

To explore the appearance and presentation level of anti-PDLIM1 AAb in the sera from OC patients and healthy controls, the indirect ELISA was performed for the measurement of anti-PDLIM1 autoantibody in two datasets (training and validation). We first tested the autoantibody in 61 OC sera and 61 age-matched healthy control sera in the training dataset. The result showed that anti-PDLIM1 autoantibody not only appeared in the sera from OC patients but also was significantly higher (*p*<0.0001) in OC sera than that in healthy control sera (**Figure 3A**). Then, we further validated the result from the training dataset with larger sample size in the validation dataset including 121 sera from patients with OC, 181 sera from patients with ovarian benign diseases, and 121 sera from healthy controls. Significant differences in the titer of anti-PDLIM1 AAb were observed between the OC group and each of two control groups (healthy controls and ovarian benign controls) (*p*<0.0001); the statistical difference still appeared when we combined two control groups into one group, without statistical difference between healthy control group and benign controls group (**Figures 3B, C****)**. There is no difference detected between early and late stage of OC on the expression of anti-PDLIM1 AAb (**Figures 3D**).

### Value of Anti-PDLIM1 Autoantibody in Diagnosis and Differential Diagnosis of OC

The performances of anti-PDLIM1 AAb in training and validation datasets were determined by comparing the parameters that can reflect the diagnostic value of OC. Based on the ROC analysis, the Youden index (YI), sensitivity, specificity, false positive rate (FPR), false negative rate (FNR), positive predictive value (PPV), and negative predictive value (NPV) were analyzed among different groups (**Table 3**). As shown in **Figures 3E** **, F**, the areas under the curve (AUC) for identifying OC from healthy controls in training and validation datasets were 0.765 (95% CI: 0.680–0.850) and 0.740 (95% CI: 0.678–0.802), respectively. In the differential diagnosis of OC in the validation dataset (**Figure 3G**), anti-PDLIM1 AAb had the AUC of 0.757 (95% CI: 0.702–0.812). When we combined all healthy controls and ovarian benign controls from both training and validation datasets, the AAb showed the AUC of 0.730 (95% CI: 0.683–0.776) for distinguishing all OC from all controls (**Figure 3H**). The AUCs for identification of OC with early stage (I+II) and late stage (III+IV) were 0.756 (95% CI: 0.666–0.845) and 0.772 (95% CI: 0.710–0.834), respectively (**Figures 3I, J****)**, without significant difference.

**Table 3:** Diagnostic value of anti-PDLIM1 AAb to OC.

Subjects	AUC	*p*	Se (%)	Sp (%)	YI	FPR (%)	FNR (%)	PPV (%)	NPV (%)
OC *vs.* Healthy	0.740	<0.0001	35.5	90.1	0.3	9.9	64.5	78.2	58.3
OC *vs.* Benign	0.757	<0.0001	33.9	90.6	0.2	9.4	66.1	78.3	57.8
OC *vs.* (Healthy+Benign)	0.730	<0.0001	31.9	90.1	0.2	9.9	68.1	76.3	57.0
Early *vs.* Healthy	0.756	<0.0001	40.6	90.1	0.3	9.9	59.4	80.4	60.3
Late *vs.* Healthy	0.772	<0.0001	39.5	90.1	0.3	9.9	60.5	80.0	59.8
Early *vs.* Benign	0.748	<0.0001	40.6	90.6	0.3	9.4	59.4	81.2	60.4
Late *vs.* Benign	0.761	<0.0001	33.3	90.6	0.2	9.4	66.7	78.0	57.6
Early *vs.* (Healthy+Benign)	0.739	<0.0001	40.6	90.7	0.3	9.3	59.4	81.4	60.4
Late *vs.* (Healthy+Benign)	0.755	<0.0001	33.3	90.1	0.2	9.9	66.7	77.1	57.5

OC, ovarian cancer; Se, sensitivity; Sp, specificity; YI, Youden Index; FPR, false positive rate; FNR, false negative rate; PPV, positive predictive value; NPV, negative predictive value.

**Figure 4:**
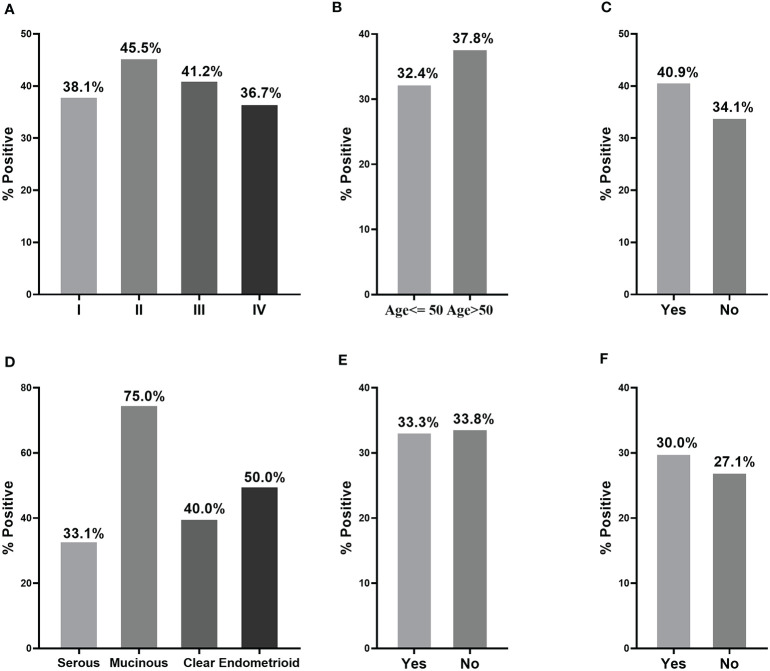
The performance of anti-PDLIM1 AAb in different subgroups of OC patients. The frequency of anti-PDLIM1 AAb among subgroups of OC patients based on stage **(A)**, age **(B)**, family tumor history **(C)**, histological type **(D)**, lymph node metastasis **(E)**, and distant metastasis **(F)**.

When a cut-off value was defined as the corresponding point to maximum of Youden index at specificity of 90%, the frequency of anti-PDLIM1 AAb in the different groups were calculated. Among 182 OC sera, 35.7% (65/182) of sera showed positive reaction to PDLIM1 (**Table 4**), which was significantly higher than that in healthy control sera (9.9%, 18/182) and ovarian benign control sera (12.2%, 22/181). Obviously, anti-PDMIL1 AAb had a certain ability to distinguish OC from normal and ovarian benign diseases. In another word, the anti-PDLIM1 AAb seemed more specific to OC across ovarian benign and malignant diseases. To evaluate the performance of anti-PDLIM1 AAb in different subgroups of OC, we divided OC patients into different groups by clinical stage, age, family tumor history, lymph node metastasis, distant metastasis, and histological types, and no significant differences were found across these subgroups (**Figure 4**). Although the positive rate of anti-PDLIM1 AAb in ovarian mucinous adenocarcinoma was as high as 75% (3/4), the sample size was too small to make a difference (*p*>0.05, Fisher’s exact test).

**Table 4:** Diagnostic performance of anti-PDLIM1 AAb, CA125, and the combination of them.

Types	Positive, n/N	%	*p*
Anti-PDLIM1+CA125	95/120	79.2	<0.0001^a^
Anti-PDLIM1	65/182	35.7	
CA125	74/120	61.7	0.003^a^
Early (Anti-PDLIM1+CA125)	11/12	91.7	0.080^b^
Late (Anti-PDLIM1+CA125)	53/63	77.8	
Healthy (Anti-PDLIM1)	18/182	9.9	<0.0001^c^
Benign (Anti-PDLIM1)	22/181	12.2	<0.0001^c^

The cut-off was determined by the maximum Youden index with specificity of 90%.

^a^The frequency of OC patients with positive reaction to both anti-PDLIM1 AAb and CA125 compared with the frequency of only anti-PDLIM1 AAb or CA125 positive.

^b^The frequency of OC patients with early stage compared to that with late stage.

^c^The frequency of OC patients with positive reaction to anti-PDLIM1 AAb compared with the frequency of healthy or ovarian benign controls with positive reaction to anti-PDLIM1 AAb.

### Combination of Anti-PDLIM1 Autoantibody and CA125 for Detecting OC

We used testing data from a total 182 OC sera and total 182 healthy control sera for evaluating the diagnostic value of the combination of anti-PDLIM1 AAb and CA125. As mentioned above, 35.7% (65/182) of OC sera was found to have positive reactivity to PDLIM1. Among 182 OC sera, 120 of OC patients had the test result of CA125; 61.7% (74/120) of OC sera showed positive reaction (>35 U/ml) of CA125 (**Table 4**). When combing anti-PDLIM1 AAb with CA125, the positive rate increased to 79.2% (95/120), which was significantly higher than anti-PDLIM1 AAb or CA125 alone (*p*<0.001). The combination of the two biomarkers yielded the AUCs of 0.846 (95% CI: 0.797–0.896) and 0.841 (0.794–0.887) for discriminating OC from healthy controls or all controls (healthy + benign), respectively (**Figures 3K, L****)**. The combination identified 91.7% (11/12) early-stage (I+II) OC and 77.8% (53/63) late-stage (III+IV) OC (**Table 4**). Moreover, anti-PDLIM1 AAb identified 15% (18/120) of OC patients with negative CA125.

## Discussion

Due to the heterogeneous attribute, most of ovarian cancer (OC) is insidious and painless in the early stage. Thus, there are only less than 25% of patients with OC detected at the early stage (I+II), and more than 75% of patients with OC are found at the late stage (II+IV) (23). Although the 5-year survival of OC patients has improved owing to advanced treatments, the overall cure rate still remains lower than 30% (24). The transvaginal ultrasonography and cancer antigen (CA) 125 are the most commonly used diagnostic tests for OC; however, they are not specific for OC and could not reflect the evidence of decreased mortality for OC (25, 26). At present, the development of the strategy for early detection of OC is imperative to reduce the mortality of OC. Therefore, with the advantages of stability and convenient detection, a blood-based biomarker such as autoantibodies holds promising interests for identifying individuals and developing strategies of early detection (27, 28). In this study, we tried to explore anti-PDLIM1 AAb as a potential biomarker for the detection of OC. Using different methods (IHC, ELISA, bioinformatics analysis) for validation, the anti-PDLIM1 AAb showed good diagnostic potential as a biomarker for the detection of OC.

To the best of our knowledge, this is the first study to explore the diagnostic value of anti-PDLIM1 AAb for the detection of OC. In a study performed by Hong *et al*., anti-PDLIM1 AAb was detected in 14 out of the 36 sera (38.9%) from patients with a pancreatic adenocarcinoma, while it was only observed in 4.4% of controls (3 out of 68 subjects including 14 lung adenocarcinoma, 19 colon adenocarcinoma, and 35 healthy subjects) (18). Another study also reported that anti-PDLIM1 AAb identified breast cancer from controls with a sensitivity of 73.4% and specificity of 58.3% (9). However, there is less evidence to show the detection and diagnostic performance of anti-PDLIM1 AAb in OC patients. From our findings, anti-PDLIM1 AAb could distinguish OC from healthy controls with the AUCs of 0.765 and 0.740 in the training and validation datasets, and 0.757 for distinguishing OC from benign controls. It could identify 35.7% (65/182) of OC at the specificity of 90.1%. Even though we combined both healthy and benign controls, the AAb still has the AUC of 0.730 with the sensitivity of 31.9% and specificity of 90.1% to discriminate OC. Therefore, this study holds some advantages. First, we measured the expression of anti-PDLIM1 AAb not only in the sera of OC patients and healthy subjects but also in the sera of patients with ovarian benign diseases. Second, the design of two different groups of controls makes our results more dependable. Third, both training and validation datasets in which the sera were from different hospitals could further make a solid conclusion. Last but not least, since the elevated anti-PDLIM1 AAb in OC sera was in line with the high expression of PDLIM1 protein in OC tissue, it was speculated that strong immune response of PDLIM1 AAb in OC patients might be triggered by the high expression of PDLIM1 protein in OC tissues. Consequently, anti-PDLIM1 AAb has great potential as a serological marker of ovarian cancer. Further works are still needed to investigate its potential utility for clinical detection.

Moreover, PDLIM1 plays an important role in the process of tumorigenesis (14, 29, 30). It serves as an important regulator for breast cancer cell migration and metastasis, and the increased expression of PDLIM1 contributes to the progression of breast cancer (17). In addition, a study indicated that PDLIM1 could promote proliferation and suppress apoptosis of chronic myeloid leukemia cells, having an oncogenic role to chronic myeloid leukemia (29). Huang’s study showed that PDLIM1 is significantly downregulated in the tissues of metastatic hepatocellular carcinoma (HCC), suggesting that PDLIM1 may be a potential prognostic marker for metastatic hepatocellular carcinoma (16). Ahn et al. reported that PDLIM1 with the interaction to neurotrophin receptor p75 as a mediator of glioma invasion could provide therapeutic strategies for patients with glioblastoma (31). From the IHC analysis in this study, the PDLIM1 protein was highly expressed in OC tissues, while it was not expressed in adjacent or normal ovarian tissues. Based on the aforementioned evidence as well as the results of our current study, we could confirm that PDLIM1 is closely associated with cancers, and thus it may be a tumor-associated antigen in ovarian cancer.

At present, the detection of AAbs attracted much attention to complement CA125 for the screening of women with ovarian cancer (32–34). Since multiplex detection could improve the sensitivity and specificity, a panel with multiple biomarkers was widely reported. A study reported that a panel with four biomarkers [CA125, macrophage inhibitory factor (MIF), osteopontin (OPN), and anti-IL-8 autoantibodies] could identify 82.0% of OC with early stage compared to 65% with CA125 alone (35). It was demonstrated that the combination of CA125, anti-SBP1, and anti-TP53 greatly improved the sensitivity and specificity of OC identification with the AUC of 0.96 (36). From this study, the combination of anti-PDLIM1 AAb and CA125 could detect 79.2% patients with OC, which is statistically higher than when using CA125 or anti-PDLIM1 AAb alone. In addition, the novel AAb identified in our study could identify 15% (18/120) of patients with negative CA125. Therefore, our findings may provide a novel biomarker for OC detection as a complementary tool to CA125. However, there are limitations in this study. Firstly, the study is a case-control study; further validations in a prospective research work are required to confirm the diagnostic value of anti-PDLIM1 AAb for OC detection based on a large sample size. Secondly, anti-PDLIM1 AAb is not fully specific to OC; it was also detected in breast cancer and pancreatic cancer. Further works are needed to explore the mechanism of PDLIM1 in OC.

In summary, our findings indicated that anti-PDLIM1 AAb was elevated in OC patients compared with healthy controls, which was consistent with the high expression of its corresponding antigen in OC tissues. Anti-PDLIM1 AAb could distinguish OC patients from both healthy subjects and ovarian benign cases, and it showed a good performance especially when combined with CA125. Therefore, anti-PDLIM1 AAb may be used as a potential biomarker for OC detection, and it could improve the sensitivity in identifying OC by the combination with CA125.

## Data Availability Statement

The raw data supporting the conclusions of this article will be made available by the authors, without undue reservation.

## Ethics Statement

The studies involving human participants were reviewed and approved by the Ethics Committee of Zhengzhou University. The patients/participants provided their written informed consent to participate in this study.

## Author Contributions

XW: Design of the study, conceptualization, writing—review and editing. JZ: Writing—review and editing. CQ: Data collection and analysis, methodology, conducting study, writing—original draft. YD: Conducting study and writing—review and editing. BW: Visualization and writing—review and editing. JS: Statistical analysis. PW: Experiments. HY: Experiments. LD: Data analysis. All authors contributed to the article and approved the submitted version.

## Funding

This study was funded by the Major Project of Science and Technology in Henan Province (No. 161100311400).

## Conflict of Interest

The authors declare that the research was conducted in the absence of any commercial or financial relationships that could be construed as a potential conflict of interest.

## Publisher’s Note

All claims expressed in this article are solely those of the authors and do not necessarily represent those of their affiliated organizations, or those of the publisher, the editors and the reviewers. Any product that may be evaluated in this article, or claim that may be made by its manufacturer, is not guaranteed or endorsed by the publisher.
